# Identification of methylation states of DNA regions for Illumina methylation BeadChip

**DOI:** 10.1186/s12864-019-6019-0

**Published:** 2020-03-05

**Authors:** Ximei Luo, Fang Wang, Guohua Wang, Yuming Zhao

**Affiliations:** 1 School of Computer Science and Technology, Harbin Institute of Technology, Harbin, China; 2 Information and Computer Engineering College, Northeast Forestry University, Harbin, China

**Keywords:** HM450K, EPIC, DNA methylation states and DNA regions

## Abstract

**Background:**

Methylation of cytosine bases in DNA is a critical epigenetic mark in many eukaryotes and has also been implicated in the development and progression of normal and diseased cells. Therefore, profiling DNA methylation across the genome is vital to understanding the effects of epigenetic. In recent years the Illumina HumanMethylation450 (HM450K) and MethylationEPIC (EPIC) BeadChip have been widely used to profile DNA methylation in human samples. The methods to predict the methylation states of DNA regions based on microarray methylation datasets are critical to enable genome-wide analyses.

**Result:**

We report a computational approach based on the two layers two-state hidden Markov model (HMM) to identify methylation states of single CpG site and DNA regions in HM450K and EPIC BeadChip. Using this mothed, all CpGs detected by HM450K and EPIC in H1-hESC and GM12878 cell lines are identified as un-methylated, middle-methylated and full-methylated states. A large number of DNA regions are segmented into three methylation states as well. Comparing the identified regions with the result from the whole genome bisulfite sequencing (WGBS) datasets segmented by MethySeekR, our method is verified. Genome-wide maps of chromatin states show that methylation state is inversely correlated with active histone marks. Genes regulated by un-methylated regions are expressed and regulated by full-methylated regions are repressed. Our method is illustrated to be useful and robust.

**Conclusion:**

Our method is valuable for DNA methylation genome-wide analyses. It is focusing on identification of DNA methylation states on microarray methylation datasets. For the features of array datasets, using two layers two-state HMM to identify to methylation states on CpG sites and regions creatively, our method which takes into account the distribution of genome-wide methylation levels is more reasonable than segmentation with a fixed threshold.

**Electronic supplementary material:**

The online version of this article (10.1186/s12864-019-6019-0) contains supplementary material, which is available to authorized users.

## Background

Methylation of DNA cytosine residues at carbon 5 (5meC), a common epigenetic mark in many eukaryotes, is often found in the CpG and CpHpG (H = A, T, C) sequence context. Methylation modification has various biological functions. Interactions between transcription factors (TFs) and methylated DNA are considered to play an important role in regulating gene expression [1–4]. DNA methylation of gene regulatory elements, such as promoters and enhancers, are generally considered to be incompatible with activated gene expression [5, 6]. However, DNA methylation levels of CpGs increase in the gene bodies of actively transcribed genes in plants and mammals [7–9]. As one of the popular research areas in gene regulation, DNA methylation is also considered to be involved in the pathogenesis of a number of tumors [10].

With the development of biological technology, large-scale DNA methylation profiling has been generated from different sequencing and microarray techniques, such as WGBS [11], RRBS (reduced representation bisulfite sequencing) [12], HM450K [13] and EPIC [14]. Analysis of DNA methylation process mainly includes the data normalization and identification of differentially methylated sites or regions. Many pipelines have been developed to solve these two issues. QN (quantile normalization), ASMN (all sample mean normalization) and PBC (peak-based correction) are often used to normalize the Illumina methylation array data [15–17]. ChAMP is a pipeline designed for HM450K chip analysis [18]. To detect the differentially methylated regions, Martin J. Aryee developed a flexible and comprehensive R package named Minfi [19]. Identifying DNA methylation status is also important for understanding its function. DMRcate was designed to identify differentially methylated regions for replicated methylation measurements from the Illumina HM450K BeadChip [20]. At present, the fixed threshold is used to determine the state of DNA methylation. Generally, if the DNA methylation level at a CpG site is higher than 80%, then it is classified as methylated, less than 20% as un-methylated and others as partially methylated [21–23]. Another study considered methylation levels below 60% as low methylation [1]. Katherine E. Varley sets more than 90% as fully methylated sites and less than 10% as unmethylated sites [24].

HMM-Fisher, which was designed to identify differential CpG sits on bisulfite sequencing data, can dynamically recognize the methylation states [25]. The Hidden Markov Model was used to divide the CpG sites into fully methylated site, lowly methylated site and unmethylated site. Strong spatial correlation is a dominant feature shown in the DNA methylation data and DNA methylation is regulated in longer genomic regions [19, 26]. Identification of DNA methylation state on DNA regions is more meaningful than identification of DNA methylation state at an individual site. MethylSeekR was designed to identify the active regulatory regions from WGBS data and divide the genomic regions into partially methylated, low-methylated and unmethylated regions [27].

Here, we developed a new method to recognize the methylation states for individual CpG site and genomic region from the methylome data generated by HM450K and EPIC. In H1-hESC and GM12878 cell lines, our method identified numerous sites and genomic regions with full, median and un-methylation. By comparing MethylSeekR result from the same cell lines, the full methylation regions and unmethylation regions have very high coincidence rate.

## Methods

### Data description and preprocessing

The DNA methylation datasets generated from Illumina HM450K array, EPIC array platform and WGBS were downloaded from the Encyclopedia of DNA Elements (ENCODE) project and GEO Datasets Database (see Additional file 1). We processed the HM450K and RPIC data using the ‘minfi’ Bioconductor package [19]. Probes were excluded if the detection *p*-value greater than 0.05. The target CpG sites annotated to the sex chromosomes or common SNPs were removed from subsequent analysis. The methylation level of each site was measured as beta-value which was the ratio of signal compared with the sum of the methylated and unmethylated probes.

The WGBS data of H1-hESC and GM12878 cell lines were performed in two biological replicates. First, we merged the two sets of methylation data by summing the read counts. At each CpG site the methylation level was calculated as the ratio of the counts of methylated reads to total reads. Then MethylSeekR was used to identify the UMRs (unmethylated regions), LMRs (low-methylated regions) and PMDs (partially methylated domains) on the WGBS datasets. The other genomic regions were treated as FMRs. The Pearson correlation coefficients of methylation level between WGBS and HM450K datasets for two cell lines and between EPIC and WGBS datasets for GM12878 cell line were calculated on the common CpG sites for different platform.

### Identification of methylation states on CpG sites

In the HM450K and EPIC, the methylation level distribution of CpG site was bimodal (one peak corresponding to the unmethylated sites and the other to the methylated sites) and ranging from 0 to 1. The important feature of DNA methylation is that the DNA methylation levels of adjacent CpG sites were very similar in many regions and they also can be very different in some regions. The Markov models can be modeled by setting a high and low transition probability respectively. And there were less middle-methylated CpG sites compared with methylated and un-methylated sites. Therefore, two-state HMM model was used to emulate the DNA methylation states and the CpG sites were grouped into two classes, high-methyl and low-methyl. Then we checked the DNA methylation levels of high-methyl and low-methyl sites. If the methylation level distributions were bimodal, we used the two-state HMM again to divide low-methyl sites into UMSs and MMSs and divide high-methyl sites into FMSs and MMSs. If the methylation level distributions show only one peak, we regard the low-methyl sites as UMSs, and the high-methyl sites as FMSs.

### Identification of methylation states on genomic regions

**Fig. 1:**
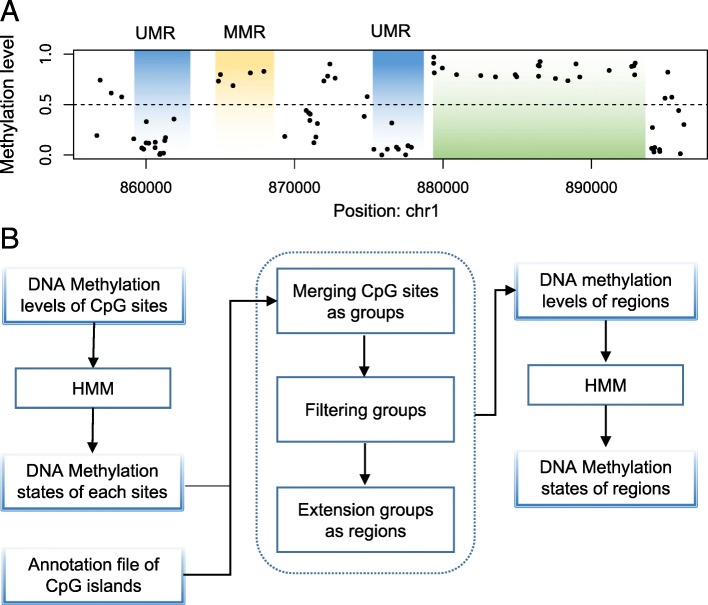
The process of identification of DNA methylation states. (**a**) An example of recognized DNA regions. (**b**) The process of DNA methylation states identification

In this study, we tried to segment the DNA genomic regions as UMRs (unmethylation regions), MMRs (median-methylated regions) and FMRs (full-methylated regions) based on the sparse DNA methylation datasets generated from HM450K and EPIC. Figure 1A showed that our method can identify some UMRs, MMRs and FMRs in H1-hESC cell line. The CpGs are un-methylated in the UMRs, and the methylation levels in FMR are always higher than MMRs. As HM450K only covers over 480,000 CpGs (2% of CpGs in whole genome) and EPIC covers 850,000 CpGs, the HM450K and EPIC datasets are relatively sparse. We set up some conditions to group the CpGs into genomic regions.

The strategy was shown in Fig. 1B. In the first step, we merged the CpG sites based on the gap and methylation states between adjacent CpGs. Obviously, the CpG densities of CpG islands and other regions are different, so the gap distances should have different threshold. Additional file 2 showed the distribution of gap distances between adjacent probes in HM450K and EPIC datasets both within and outside of CpG islands. According to the distribution, we grouped the CpG sites within 300 bp of the CpG island into genomic regions in HM450K and EPIC datasets. For the outside regions of CpG island, thresholds for gap distances are 11,300 bp in HM450K and 4800 bp in EPIC datasets. Since few CpG sites were not enough to support a regional methylation state, genomic regions less than three CpG sites were filtered. In the third step, for the extension, the same gap cutoff was used to merge the regions again to avoid the fragmentation of the region only with a change in the methylation state of one or two CpGs. Using the same HMM model of CpG sites methylation states, the genomic regions were identified to UMRs, MMRs and FMRs based on the average methylation levels.

### Using HMM to model DNA methylation

HMM was used to identify DNA methylation states. The identification of high-methyl and low-methyl CpG sites was used as an example to explain the model. The hidden states of CpGs’ methylation have both high-methyl and low-methyl states. For *k* CpGs, the hidden methylation state sequence is referred as:

$$ H=\left\{{h}_1,\cdots, {h}_{k-1},{h}_k\right\}. $$

For *k* CpGs, the methylation level sequence is used as observed sequence and referred as:

$$ O=\left\{{o}_1,\cdots, {o}_{k-1},{o}_k\right\}. $$

To train the HMM, the initial transition probability and the emission distribution of each individual hidden state need to be initialized based on prior information. Baum-Welch algorithm [28] is used to find local optima for parameters. According to the bimodal distribution of CpG methylation, the cutoff of DNA methylation was set as 0.6 empirically. The low-methyl and high-methyl states are referred as *L*_*me*_ and *H*_*me*_, respectively. Depending on the methylation level, the CpG sites were initially divided into two groups:

1 $$ {h}_i=\left\{\begin{array}{ccc}{L}_{me},& if& {o}_i>0.6\\ {}{H}_{me},& if& {o}_i\le 0.6\end{array}\right. $$

The transition probability was initialized by the frequency of the methylation’s changes between the adjacent regions (or sites):

2 $$ P\left({h}_i|{h}_{i-1}\right)=\left[\begin{array}{cc}P\left({h}_i={L}_{me}|{h}_{i-1}={L}_{me}\right)& P\left({h}_i={L}_{me}|{h}_{i-1}={H}_{me}\right)\\ {}P\left({h}_i={H}_{me}|{h}_{i-1}={L}_{me}\right)& P\left({h}_i={H}_{me}|{h}_{i-1}={H}_{me}\right)\end{array}\right] $$

The normal distribution was used to approximate the emission distributions. The variances and means of these distributions were estimated based on two groups methylation levels, respectively. Hence, the truncated normal distribution was used as the initial emission probability:

3 $$ {o}_i\mid {h}_i=\left\{\begin{array}{cc} Tnormal\left({\mu}_{L_{me}},{\theta}_{L_{me}}^2\right)\kern0.5em if& {h}_i={L}_{me}\\ {} Tnormal\left({\mu}_{H_{me}},{\theta}_{H_{me}}^2\right)\kern0.5em if& {h}_i={H}_{me}\end{array}\right. $$

For each group of methylated regions (or sites), the joint probability is:

4 $$ P\left(O,H\right)=P\left(O|H\right)P(H)=P\left({h}_1\right)P\left({o}_1|{h}_1\right)\prod \limits_{i=2}^KP\left({h}_i|{h}_{i-1}\right)P\left({o}_i|{h}_i\right) $$

Using Baum-Welch algorithm, the maximum likelihood estimate of the parameters of the Hidden Markov model were found. Based on the trained model, methylation states of sites (or regions) were predicted by Viterbi algorithm [29].

## Results

### DNA methylation states of H1-hESC and GM12878 cell lines

Method descripted above was used to identify methylation states of CpG sites and genomic regions in H1-hESC and GM12878 cell lines. The identified sites and regions are summarized in the Table 1. We found that in each sample, 30–40% of identified CpGs were UMSs and only 2–10% of identified regions were UMRs. This distinction occurred due to the fact that the un-methylated CpGs are always located in short CpG islands which have high frequencies of CpG dinucleotides. In H1-hESC cell line the identified UMSs account for 37% which is more than GM12878 (HM450K: 36.74%, EPIC: 31.67%) and the identified MMSs account for 13.45% less than GM12878 (HM450K: 38.93%, EPIC: 41.19%). FMRs account for 49.54% in H1-hESC higher than GM12878 (HM450K: 24.33%, EPIC: 27.14%). Methylation levels genome-wide in H1-hESC are higher than that in GM12878.

**Table 1:** The coverage of the identified sites and regions

Cell_type	UMSs	MMSs	FMSs	UMRs(bp)	MMRs(bp)	FMRs(bp)
H1-hESC(HM450K)	168,926	61,414	226,136	19,208,170	45,047,177	200,680,915
GM12878(HM450K)	168,035	178,077	111,277	11,817,492	133,011,338	115,296,960
GM12878(EPIC)	258,499	336,134	221,514	11,178,153	264,470,336	99,186,974

**Fig. 2:**
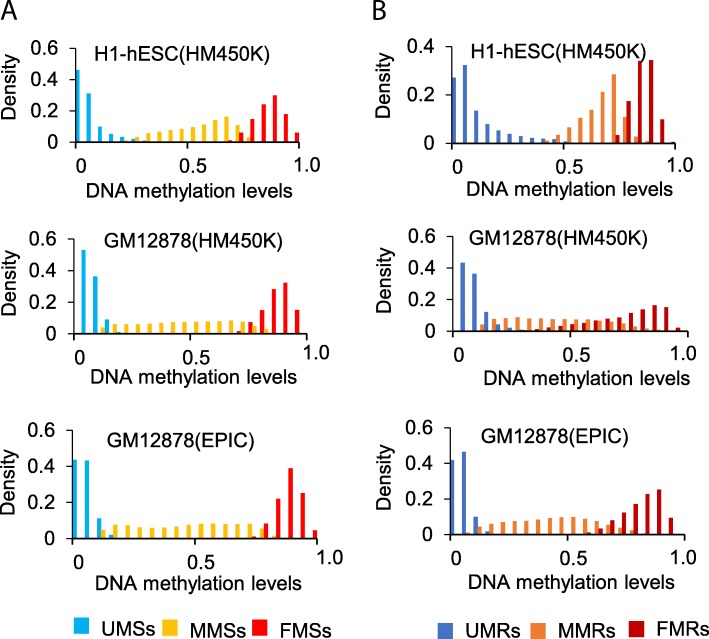
The distribution of DNA methylation level of sites and regions in different methylation states in two cell lines. (**a**) The distribution of DNA methylation level of CpG sites. (**b**) The distribution of DNA methylation level of regions

As shown in Fig. 2, the identified CpG sites and regions have their own special DNA methylation distribution. For CpG sites, the cutoff separating UMSs from MMSs is around 0.2, and the cutoff separating MMSs from FMSs is around 0.75 in GM12878. For CpG sites, the cutoff separating UMSs from MMSs is around 0.2, and the cutoff separating MMSs from FMSs is around 0.88 in H1-hESC. Such difference was due to the fact that the genome-wide methylation level in H1-hESC cell line is higher than GM12878. This approach takes it into account that the changes and distribution of methylation levels of surrounding CpG sites by using HMM models. It can be viewed as a soft segmentation which is more reasonable when comparing with the segmentation with a fixed threshold. The threshold between UMSs and MMSs methylation levels of in H1-hESC is higher than the threshold in GM12878. For regions, the threshold between the methylation levels of UMRs and MMRs is around 0.5 in H1-hESC which is also higher than that in GM12878. The distribution of DNA methylation on MMRs in H1-hESC is also higher than in GM12878.

### High consistency with the result of MethylSeekR

To the best of our knowledge, our method is the only available method to identify methylation states for HM450K and EPIC datasets. Therefore, it cannot be compared with existing methods designed for array datasets. To evaluate our method, we compared our result with the regions identified by MethylSeekR on WGBS datasets in the same cell lines. However, the definition of methylation states is different between our method and MethylSeekR. There is no definition of FMRs in MethylSeekR, but in the first step of MethylSeekR to segment WGBS as regions is filtering the region containing continuous CpG sites with methylation levels higher than a cutoff of methylation level. These regions are regarded as FMRs. The condition is looser than the FMRs in our method. The low-methylated regions in MethylSeekR lie in intergenic and intronic regions distal to transcription start sites. Because the array design only contains few CpGs in these regions, our method cannot identify low-methylated regions. Notably, we can identify a new methylation state with moderate methylation levels with our model.

**Fig. 3:**
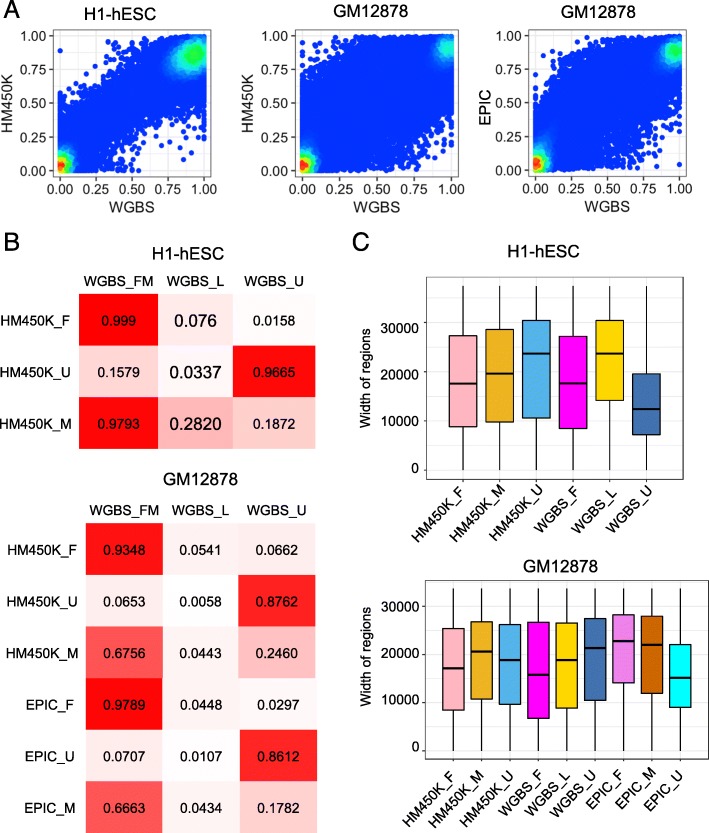
Comparison with MethylSeekR. (**a**) The correlation coefficients between WGBS and array datasets. (**b**) The overlap between the regions identified by our method and MethylSeekR. (**c**) Box plots of the widths of identified regions

First, correlation of datasets generated from two different platforms of H1-hESC and GM12878 cell lines were calculated. Pearson correlation coefficient (0.9640) between HM450K and WGBS datasets in H1-hESC cell line is higher than the coefficient (0.9059) between HM450K and WGBS datasets in GM12878 and the coefficient (0.9134) between EPIC and WGBS datasets in GM12878 (Fig. 3A). And then, methylation states of WGBS in H1-hESC and GM12878 cell lines were identified by MethylSeekR. As shown in Fig. 3B, the FMRs and UMRs recognized by our method are highly coincident with the regions identified by MethylSeekR on WGBS dataset. As the correlation of datasets on GM12878 is lower than H1-hESC, the coincidence of identified regions is lower than H1-hESC. In two cell lines, more than 93% FMRs and 85–96% UMRs are consistent with WGBS. Less than 8% FMRs and 16% UMRs overlap with the regions with different methylation states. Due to the definition of FMRs in MethylSeekR is looser than our method, the MMRs which are newly identified and have different methylation distribution from FMRs and UMRs overlapped with full methylation regions in MethylSeekR highly.

Next, we calculated the width of the regions, and found that regardless of datasets, the lengths of regions are mostly in a range from 10,000 to 30,000 (Fig. 3C). The UMRs we identified are longer than UMRs identified by MethylSeekR in H1-hESC. In GM12878, the FMRs and MMRs identified from EPIC are longer than the result obtained from WGBS and HM450K. And UMRs identified from EPIC are shorter than the result obtained from WGBS and HM450K. As the gap of conditions in the process to merge CpG sites are different and the CpG sites coverages are different between the EPIC and HM450K, the lengths of regions are different.

### Distribution of gene expression related with UMRs, MMRs and FMRs

**Fig. 4:**
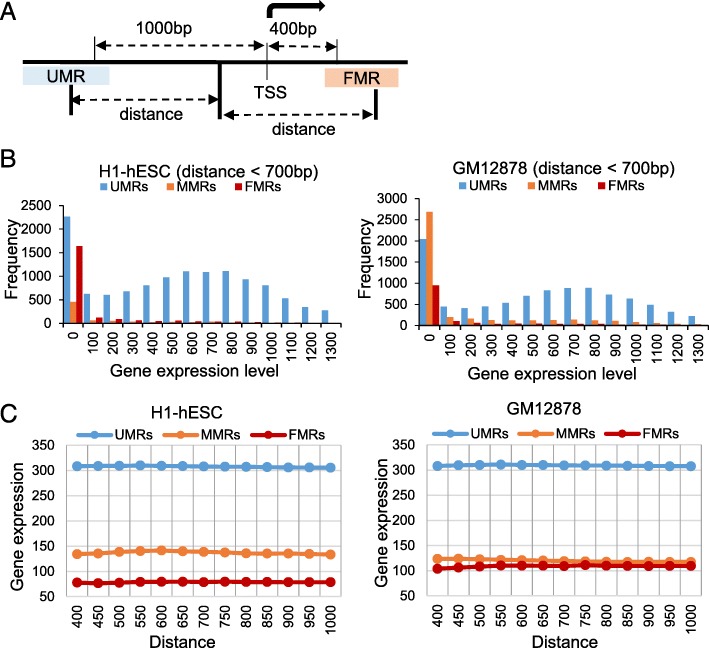
Gene expressions regulated by UMRs, MMRs and FMRs. (**a**) Definition of promoter regions and distances between promoters and UMRs, MMRs and FMRs. (**b**) The distribution of gene expressions of the genes with promoters overlapping on UMRs MMRs and FMRs. (**c**) The mean of gene expression with different distances between gene promoters and the UMRs, MMRs and FMRs

To explore gene expression pattern of the gene regulated by the UMRs, MMRs and FMRs overlapped on gene promoters, we integrated the DNA methylation states with genome-wide gene expression data from the same cell lines. When setting the promoter regions 1000 bp upstream and 400 bp downstream of transcription start sites (TSS), the distances between the middle of promoters (300 bp upstream of TSS) and the middle of identified regions were calculated (Fig. 4A). If the distance is less than the cutoff, the gene is regarded as being regulated by the identified regions. When the distance cutoff was 700 bp, we retrieved the target genes regulated by UMRs, MMRs and FMRs in H1-hESC and GM12878 and plotted the distribution of gene expression (Fig. 4B). In two cell lines, the UMRs-regulated genes showed apparent up regulation and the MMRs- and FMRs-regulated genes showed no expression. In H1-hESC, 72.82% genes with promoters overlapped on UMRs have gene expression higher than 300. Besides, only 30% genes with promoters overlapped on MMRs and 17.81% genes with promoters overlapped on FMRs are higher than 300 in gene expressions in H1-hESC. In GM12878, the gene expressions higher than 300 contain 70.94, 28.47 and 23.65% genes with promoters overlapped on UMRs, MMRs and FMRs, respectively.

After adjusting the cutoff from 400 to1000bp, we retrieved the mean of gene expressions regulated by identified regions. Figure 4C showed the UMRs-regulated genes up-regulated expression apparently. MMRs-regulated genes are higher than FMRs-regulated genes in both two cell lines. DNA methylation typically acts to repress gene transcription in a gene promoter by blocking transcription factors binding. Therefore, UMRs overlapped gene promoters show high activity. FMRs are the opposite. MMRs are between FMRs and UMRs. The quantitative relationship between gene promoter methylation and gene expression needs further exploration.

### Different methylation states have different chromatin states

**Fig. 5:**
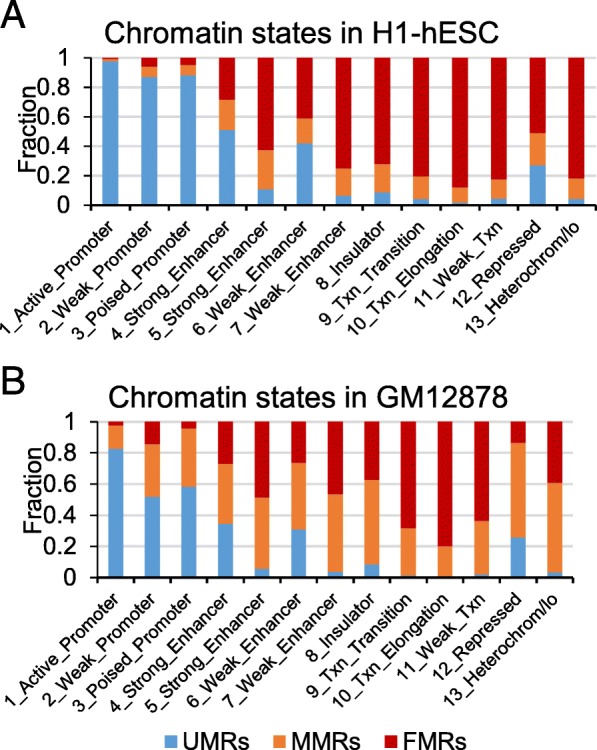
Chromatin state segmentations of identified regions. (**a**) The chromatin state segmentations in H1-hESC. (**b**) The chromatin state segmentations in GM12878

At last, we compared the identified regions with chromatin state segmentations which were learned by computationally integrating ChIP-seq data for nine factors plus input using hidden Markov model [30]. Consistent with the classical view, DNA methylation states of active promoter regions are always un-methylated, and the heterochromatin regions are high methylated (Fig. 5A). The UMRs in H1-hESC mainly occupy promoter regions, 4_strong_Enhancer and 6_weak_Enhancer, and FMRs mainly occupy 13_heterochrom_IO, 9_weak_txn, 7_weak_enhancer and 8_insulator regions. Only small amount of FMRs and MMRs occupy weak promoters and poised promoters. As shown in Fig. 5B, GM12878 has a different pattern from that in H1-hESC. Greater proportion of weak and poised promoters overlapped on MMRs and FMRs in GM12878 than H1-HESC. Overall, the methylation states of active promoter are UMRs, which mainly occupied promoter regions, 4_strong_Enhancer and 6_weak_Enhancer. However, the relationship between chromatin states and DNA methylation states is slightly different in different cell lines.

## Discussion

In this study, we presented a novel computational method for identifying DNA methylation states of CpG sites and DNA regions from HM450K and FPIC datasets by using HMM model. In our method, we combined the benefits of HMM models and DNA methylation levels context to identify methylation states dynamically. HMM-Fisher used three-state HMM to identify the methylation states of sequencing data. For methylation array, the distribution of methylation sites is not as same as bisulfite sequencing data. In array, a large number of CpG sites are distributed in both full-methylated and un-methylated states. We used two layers two-state HMM to replace three-state HMM directly. This strategy can avoid misidentifying MMSs effectively. Our method is the first one to identify DNA methylation states on array datasets. By comparing with the result from using MethylSeekR with WGBS in same cell lines, we can confirm the accuracy of the method. The advantage of our method is revealed especially when applied to the relatively sparse dataset generated from HM450K. We can infer the DNA methylation states reasonably based on a few detected CpG sites.

Our method can be viewed as a soft segmentation on DNA methylation array dataset. Generally, researchers set a fixed threshold to determine the methylation states of CpG sites or regions. However, it is unreasonable to set a fixed threshold to determine the methylation states in different cell lines. As shown in Fig. 2, thresholds are mutable in different cells. The genome-wide DNA methylation in H1-hESC cell line is higher than GM12878. In our method, the DNA methylation of MMRs in H1-hESC are higher than that in GM12878. By comparing with the result identified by MethylSeekR based on WGBS, we confirmed our method is reasonable.

This method was also applied to EPIC. On the same cell line, we predicted the DNA methylation states from the datasets generated from HM450K and EPIC array, respectively. EPIC is designed to determine more CpG sites in regions which identified as potential enhancers by FANTOM5 [31] and ENCODE. That information is not covered in HM450K array. By comparing the result from two types of arrays, FMSs and MMSs determined in EPIC are two-fold of that in HM450K and UMSs in EPIC are 1.5-fold of that in HM450K. In terms of DNA regions, identified MMRs in EPIC are two-fold of that in HM450K. It could be associated with the additional measurement regions. As the HM450K has covered more then 95% CpG islands, the UMRs regions identified are approximately the same in length. There are three outcomes due to the fact that the EPIC has more CpG sites. The cutoff of gaps in merging regions is shorter than it of HM450K. The boundaries of the identified regions are detected more accurately. The length of MMRs is shorter than HM450K. Comparing our results to MethylSeekR, EPIC is more accurate in identifying FMRs than HM450K.

## Conclusions

In this work, we introduced a two-layer two-state HMM to identify methylation states of CpG sites and regions assessed by HM450K and FPIC datasets. Our approach combined the background of genome-wide methylome, dividing the CpG sites and regions into three methylation states, avoiding a fixed threshold to recognize methylation states.

In addition, we applied this innovative approach to identify methylation states for array datasets in H1-hESC and GM12878 cell lines. This approach is evaluated by comparing with the result identified by MethylSeekR based on WGBS dataset. We also observed the methylation level distribution of the same methylation states in different cell lines are different. By comparing the expression of genes with promoters in different methylation states, the expression of genes in the UMRs is much higher than that in the FMRs and MMRs. We also observed differences in chromatin status in different methylated regions. These suggest that this novel computational method can avoid setting fixed threshold when identifying methylation states on CpG sites and regions for array datasets effectively and correctly.

## Additional files

Additional file 1: Data Sources (PPTX 38 kb)

Additional file 2: The distribution of gap distances between adjacent CpG sites (PPTX 57 kb)

## Data Availability

In this study, the methylation datasets were downloaded from ENCODE project. The accession IDs about WGBS datasets on H1-hESC and GM12878 are ENCSR617FKV and ENCSR890UQO. The accession IDs about HM450K datasets on H1-hESC and GM12878 are ENCSR000ABI and ENCSR000ACX. The accession ID about EPIC datasets on GM12878 is ENCSR135FMA.

## Abbreviations

EPIC: MethylationEPIC BeadChip
FMR: Full-methylated region
FMS: Full-methylated site
HM450K: Illumina HumanMethylation450 BeadChip
HMM: Hidden Markov model
MMR: Middle-methylated region
MMS: Middle-methylated site
RRBS: Reduced representation bisulfite sequencing
UMR: Unmethylated region
UMS: Unmethylated site
WGBS: Whole genome bisulfite sequencing
