# Supplementary material for: Identification of methylation states of DNA regions for Illumina methylation BeadChip
Source: BMC Genomics. 2020 Mar 5;21(Suppl 1):672. doi: 10.1186/s12864-019-6019-0 (PMC7057447; doi:10.1186/s12864-019-6019-0)
Supplement: Supplementary file 1 — Data Sources (PPTX 38 kb) [file 12864_2019_6019_MOESM1_ESM.pptx]

## Slide 1
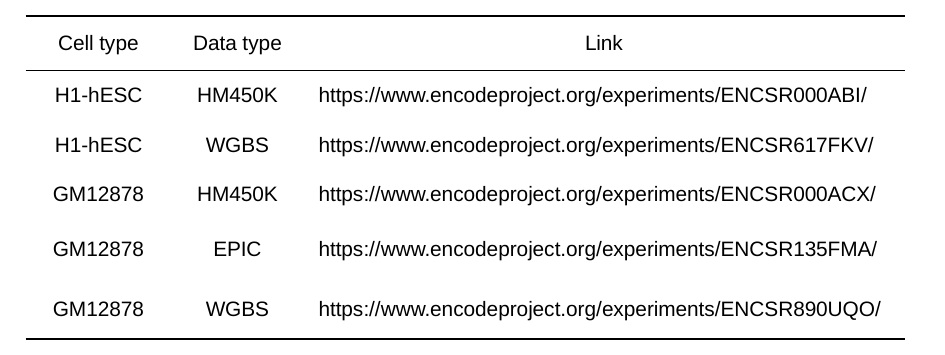

| Cell type | Data type | Link |
| --- | --- | --- |
| H1-hESC | HM450K | https://www.encodeproject.org/experiments/ENCSR000ABI/ |
| H1-hESC | WGBS | https://www.encodeproject.org/experiments/ENCSR617FKV/ |
| GM12878 | HM450K | https://www.encodeproject.org/experiments/ENCSR000ACX/ |
| GM12878 | EPIC | https://www.encodeproject.org/experiments/ENCSR135FMA/ |
| GM12878 | WGBS | https://www.encodeproject.org/experiments/ENCSR890UQO/ |
