# Supplementary material for: Identification of methylation states of DNA regions for Illumina methylation BeadChip
Source: BMC Genomics. 2020 Mar 5;21(Suppl 1):672. doi: 10.1186/s12864-019-6019-0 (PMC7057447; doi:10.1186/s12864-019-6019-0)
Supplement: Supplementary file 2 — The distribution of gap distances between adjacent CpG sites (PPTX 57 kb) [file 12864_2019_6019_MOESM2_ESM.pptx]

## Slide 1
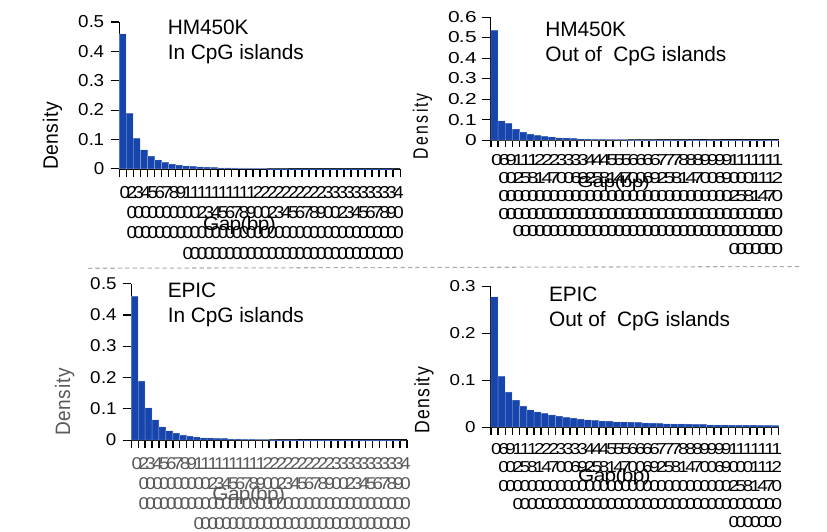

### Chart
| Category | Fraction |
|---|---|
| 0.0 | 0.535794837 |
| 6000.0 | 0.095121592 |
| 9000.0 | 0.081894786 |
| 12000.0 | 0.053879299 |
| 15000.0 | 0.039292677 |
| 18000.0 | 0.029773965 |
| 21000.0 | 0.023313473 |
| 24000.0 | 0.018556847 |
| 27000.0 | 0.015727993 |
| 30000.0 | 0.012342106 |
| 30000.0 | 0.01117889 |
| 36000.0 | 0.009393107 |
| 39000.0 | 0.007869458 |
| 42000.0 | 0.006973836 |
| 45000.0 | 0.005848847 |
| 48000.0 | 0.005117059 |
| 51000.0 | 0.004510876 |
| 54000.0 | 0.004112215 |
| 57000.0 | 0.003648021 |
| 60000.0 | 0.003047299 |
| 60000.0 | 0.002888927 |
| 66000.0 | 0.002517571 |
| 69000.0 | 0.002430193 |
| 72000.0 | 0.00211891 |
| 75000.0 | 0.001905927 |
| 78000.0 | 0.001884083 |
| 81000.0 | 0.001534572 |
| 84000.0 | 0.001485422 |
| 87000.0 | 0.001485422 |
| 90000.0 | 0.001086761 |
| 90000.0 | 0.001108605 |
| 96000.0 | 0.001141372 |
| 99000.0 | 0.000977538 |
| 102000.0 | 0.000890161 |
| 105000.0 | 0.000841011 |
| 108000.0 | 0.000813705 |
| 111000.0 | 0.000726327 |
| 114000.0 | 0.000709944 |
| 117000.0 | 0.000720866 |
| 120000.0 | 0.000633489 |
### Chart
| Category | Fraction |
|---|---|
| 0.0 | 0.459973252157576 |
| 200.0 | 0.188522092068512 |
| 300.0 | 0.103947278783511 |
| 400.0 | 0.0654640894080695 |
| 500.0 | 0.043008092477259 |
| 600.0 | 0.0305269611797735 |
| 700.0 | 0.0217317255943865 |
| 800.0 | 0.0162136385312351 |
| 900.0 | 0.012739287417399 |
| 1000.0 | 0.0102150942449113 |
| 1000.0 | 0.00811757577061395 |
| 1200.0 | 0.00714231931760732 |
| 1300.0 | 0.00571528965475204 |
| 1400.0 | 0.0050125313283208 |
| 1500.0 | 0.00414125442361268 |
| 1600.0 | 0.00377911875540066 |
| 1700.0 | 0.00319826748559525 |
| 1800.0 | 0.00278952029573218 |
| 1900.0 | 0.0023090630725598 |
| 2000.0 | 0.00225528054757781 |
| 2000.0 | 0.00143420066618621 |
| 2200.0 | 0.00082466538305707 |
| 2300.0 | 0.000394405183201208 |
| 2400.0 | 0.000215130099927931 |
| 2500.0 | 7.88810366402415e-05 |
| 2600.0 | 4.66115216510518e-05 |
| 2700.0 | 3.58550166546552e-05 |
| 2800.0 | 3.94405183201208e-05 |
| 2900.0 | 2.15130099927931e-05 |
| 3000.0 | 1.07565049963966e-05 |
| 3000.0 | 2.50985116582587e-05 |
| 3200.0 | 1.07565049963966e-05 |
| 3300.0 | 1.79275083273276e-05 |
| 3400.0 | 7.17100333093105e-06 |
| 3500.0 | 3.58550166546552e-06 |
| 3600.0 | 3.58550166546552e-06 |
| 3700.0 | 1.43420066618621e-05 |
| 3800.0 | 3.58550166546552e-06 |
| 3900.0 | 3.58550166546552e-06 |
| 4000.0 | 0.0 |HM450K
In CpG islands
HM450K
Out of CpG islands
### Chart
| Category | Fraction |
|---|---|
| 0.0 | 0.459973252157576 |
| 200.0 | 0.188522092068512 |
| 300.0 | 0.103947278783511 |
| 400.0 | 0.0654640894080695 |
| 500.0 | 0.043008092477259 |
| 600.0 | 0.0305269611797735 |
| 700.0 | 0.0217317255943865 |
| 800.0 | 0.0162136385312351 |
| 900.0 | 0.012739287417399 |
| 1000.0 | 0.0102150942449113 |
| 1000.0 | 0.00811757577061395 |
| 1200.0 | 0.00714231931760732 |
| 1300.0 | 0.00571528965475204 |
| 1400.0 | 0.0050125313283208 |
| 1500.0 | 0.00414125442361268 |
| 1600.0 | 0.00377911875540066 |
| 1700.0 | 0.00319826748559525 |
| 1800.0 | 0.00278952029573218 |
| 1900.0 | 0.0023090630725598 |
| 2000.0 | 0.00225528054757781 |
| 2000.0 | 0.00143420066618621 |
| 2200.0 | 0.00082466538305707 |
| 2300.0 | 0.000394405183201208 |
| 2400.0 | 0.000215130099927931 |
| 2500.0 | 7.88810366402415e-05 |
| 2600.0 | 4.66115216510518e-05 |
| 2700.0 | 3.58550166546552e-05 |
| 2800.0 | 3.94405183201208e-05 |
| 2900.0 | 2.15130099927931e-05 |
| 3000.0 | 1.07565049963966e-05 |
| 3000.0 | 2.50985116582587e-05 |
| 3200.0 | 1.07565049963966e-05 |
| 3300.0 | 1.79275083273276e-05 |
| 3400.0 | 7.17100333093105e-06 |
| 3500.0 | 3.58550166546552e-06 |
| 3600.0 | 3.58550166546552e-06 |
| 3700.0 | 1.43420066618621e-05 |
| 3800.0 | 3.58550166546552e-06 |
| 3900.0 | 3.58550166546552e-06 |
| 4000.0 | 0.0 |
### Chart
| Category | Fraction |
|---|---|
| 0.0 | 0.277665361759156 |
| 600.0 | 0.108073674372675 |
| 900.0 | 0.0745913347087764 |
| 1200.0 | 0.0574666278721191 |
| 1500.0 | 0.044620342450052 |
| 1800.0 | 0.0368768625791321 |
| 2100.0 | 0.032682202119593 |
| 2400.0 | 0.0290496217531609 |
| 2700.0 | 0.026164277275212 |
| 3000.0 | 0.023349468338359 |
| 3000.0 | 0.020653688127105 |
| 3600.0 | 0.0189850817330582 |
| 3900.0 | 0.0171621788428644 |
| 4200.0 | 0.0157867357914969 |
| 4500.0 | 0.014389250383537 |
| 4800.0 | 0.0134171824578109 |
| 5100.0 | 0.0127074185755347 |
| 5400.0 | 0.0117816395986528 |
| 5700.0 | 0.0110476291241249 |
| 6000.0 | 0.0103885626620113 |
| 6000.0 | 0.00988158846038548 |
| 6600.0 | 0.00890731629900016 |
| 6900.0 | 0.00844663104621841 |
| 7200.0 | 0.00817110158881306 |
| 7500.0 | 0.00731144968170837 |
| 7800.0 | 0.00689925761342997 |
| 8100.0 | 0.00674275688162373 |
| 8400.0 | 0.00625121232961258 |
| 8700.0 | 0.00582799908303797 |
| 9000.0 | 0.00556569503958807 |
| 9000.0 | 0.00507855895889541 |
| 9600.0 | 0.00480743797280855 |
| 9900.0 | 0.00452529580842547 |
| 10200.0 | 0.00442169673244106 |
| 10500.0 | 0.00438202049057469 |
| 10800.0 | 0.00419025198822056 |
| 11100.0 | 0.00403154702075508 |
| 11400.0 | 0.00376924297730519 |
| 11700.0 | 0.00357527023929182 |
| 12000.0 | 0.00333280431677511 |EPIC
In CpG islands
EPIC
Out of CpG islands
